# 962. Classification Systems & Clinical Outcomes of Axillary Burn Contracture: A Systematic Review & Meta-analysis

**DOI:** 10.1093/jbcr/irag033.540

**Published:** 2026-04-06

**Authors:** Mare G Kaulakis, Gina M Frigic, Aaron J Yeh, Christopher J Fedor, Sarah M Tepe, Francesco M Egro, Justine S Kim, Jonathan S Friedstat, Andrei Odobescu, T Justin Gillenwater

**Affiliations:** University of Pittsburgh Medical Center, Mercy Burn Center, Pittsburgh, PA; Northeast Ohio Medical University, Rootstown, OH; Northeast Ohio Medical University, Rootstown, OH; University of Pittsburgh Medical Center, Department of Plastic Surgery, Pittsburgh, PA; University of Pittsburgh Medical Center, Pittsburgh, PA; University of Pittsburgh Medical Center, Pittsburgh, PA; University of Pittsburgh Medical Center, Mercy Burn Center, Pittsburgh, PA; Massachusetts General Hospital, Boston, MA; University of Texas Southwestern Medical Center, Department of Plastic Surgery, Dallas, TX; Division of Plastic and Reconstructive Surgery, Keck School of Medicine, University of Southern California, Los Angeles, California

## Abstract

**Introduction:**

Axillary burn contracture is a common and debilitating complication of burn injuries, impairing mobility and quality of life. Multiple classification systems and treatments exist, but the optimal choice remains controversial as there is limited systematic data comparing outcomes across techniques and classifications.

**Methods:**

A systematic review was performed on surgical management of post-burn axillary contractures. PubMed, Embase, and Web of Science were searched for eligible studies that include full-text original articles with >3 human patients. Data were extracted and quality assessed independently by two reviewers with disputes settled by a third. Outcomes were synthesized qualitatively and, where feasible, meta-analyzed with subgroup analyses by contracture type.

**Results:**

Of 1196 articles screened, 64 met inclusion, encompassing 1823 patients with axillary burn contracture. Most were case series, with few comparative designs. Formal classification systems were rarely applied; when used, Kurtzman and Stern was most common. Reported surgical approaches included skin grafting (n = 327), scapular flaps (n = 117), and square flaps (n = 76), with additional series describing other local, regional, and free flap techniques. Variable reporting and inconsistent classification use limited detailed subgroup analysis.

**Conclusions:**

Local flap techniques appear to provide favorable outcomes in axillary burn contractures, with lower recurrence and better functional preservation reported in several studies compared to skin grafting. However, inconsistent use of classification systems and variable outcome reporting limit direct comparisons across studies. Standardized reporting of contracture type and surgical outcomes is needed to guide evidence-based selection of reconstructive techniques. These findings suggest that flap-based reconstructions may be advantageous, particularly for more severe contractures, though further high-quality studies are needed.

**Applicability of Research to Practice:**

This study addresses different surgical approaches for post-burn axillary contractures. The findings help clarify whether current surgical patterns align with best outcomes and inform evidence-based recommendations for managing these cases.

**Funding for the study:**

No external funding was received for this review. The review is supported by the guarantor/review team’s (non-commercial) institutional affiliations.

